# Image classification and identification for rice leaf diseases based on improved WOACW_SimpleNet

**DOI:** 10.3389/fpls.2022.1008819

**Published:** 2022-10-17

**Authors:** Yang Lu, Xinmeng Zhang, Nianyin Zeng, Wanting Liu, Rou Shang

**Affiliations:** ^1^College of Information and Electrical Engineering, Heilongjiang Bayi Agricultural University, Daqing, China; ^2^Heilongjiang Provincial Key Laboratory of Networking and Intelligent Control, Northeast Petroleum University, Daqing, China; ^3^Department of Instrumental and Electrical Engineering, Xiamen University, Xiamen, China; ^4^Artificial Intelligence Energy Research Institute, Northeast Petroleum University, Daqing, China; ^5^Sanya Offshore Oil and Gas Research Institute, Northeast Petroleum University, Sanya, China

**Keywords:** WOACW, CNN, rice leaf disease, image recognition, deep learning

## Abstract

In view of the problem that manual selection of hyperparameters may lead to low performance and large consumption of manpower cost of the convolutional neural network (CNN), this paper proposes a nonlinear convergence factor and weight cooperative self-mapping chaos optimization algorithm (WOACW) to optimize the hyperparameters in the identification and classification model of rice leaf disease images, such as learning rate, training batch size, convolution kernel size and convolution kernel number. Firstly, the opposition-based learning is added to the whale population initialization with improving the diversity of population initialization. Then the algorithm improves the convergence factor, increases the weight coefficient, and calculates the self-mapping chaos. It makes the algorithm have a strong ability to find optimization in the early stage of iteration and fast convergence rate. And disturbance is carried out to avoid falling into local optimal solution in the late stage of iteration. Next, a polynomial mutation operator is introduced to correct the current optimal solution with a small probability, so that a better solution can be obtained in each iteration, thereby enhancing the optimization performance of the multimodal objective function. Finally, eight optimized performance benchmark functions are selected to evaluate the performance of the algorithm, the experiment results show that the proposed WOACW outperforms than 5 other common improved whale optimization algorithms. The WOACW_SimpleNet is used to identify rice leaf diseases (rice blast, bacterial leaf blight, brown spot disease, sheath blight and tungro disease), and the experiment results show that the identification average recognition accuracy rate reaches 99.35%, and the F1-score reaches 99.36%.

## 1 Introduction

Rice is one of the most important rations for the people. In China, rice is planted on about 25% of the country’s arable land (Sethy et al., 2020), which is second only to wheat and corn. According to statistics, rice is the most widely recognized nutritious food in Asia and the food source of more than half of the world’s population, so the quality and yield of rice have been highly valued by society and even the world. However, frequent plant diseases change the original morphology of plants to destroy its important functions. For rice, its growth process is susceptible to rice blast, bacterial leaf blight, brown spot, sheath blight, tungro disease and other diseases. For this reason, the disease identification is urgent and important in the stage of rice growth.

Some traditional crop diseases are generally identified by plant protection experts which mainly rely on artificial visual. It will not only waste time and human resources, but also obtain low recognition accuracy, strong subjectivity and easy to be affected by other factors. During the 3-6 months of rice growth, agricultural workers often apply chemical controls to protect rice at different stages of rice growth, including disinfection with chemical agents before planting and spraying pesticides during susceptible growth stages. However, the irregular use of pesticides will cause adverse consequences for the environment and human health (Wu et al., 2017). Therefore, how to quickly and accurately obtain crop disease information, so as to rationally use pesticides has become the key problem.

With the continuous development of image processing technology, the diagnostic accuracy of crop diseases has been improved and the identification results can be the scientific basis for the scientific control of crops. Till now, some achievements have been reported. Su B et al. performed machine vision processing on the strawberry images. First, the median filtering was used to remove noise, and then five classic edge segmentation detection algorithms were employed to segment the strawberry outline. Finally the area, perimeter and color characteristics of the segmented target image were evaluated, which contributed to the development of strawberry automatic grading equipment to a certain extent (Su and Hua, 2018). Tewari et al. (2020) processed rice pest and disease images *via* using an image segmentation method based on chromatic aberration and identified the diseased parts which assisted in the development of a real-time variable speed chemical spray system. Park et al. (2018) applied multispectral image pattern recognition technology to identify pests and diseases, and the accuracy was up to 99.00%.

Over the past few years, the research of machine learning in the field of image recognition had been developed. Some researchers used support vector machine (SVM) to detect and classify images (Kaur et al., 2019; Liu et al., 2020). Zhang et al. (2018a) used the K-means clustering algorithm to identify four cucumber leaf disease images and three apple leaf disease images. Zhao et al. (2020) proposed an image segmentation method based on fuzzy C-means (FCM), where the initial parameters are optimized *via* particle swarm optimization (PSO) algorithm. Compared to the classical FCM, the improved FCM has smaller objective function values, sharper image segmentation boundaries, and good noise immunity. Although machine learning has a good image classification effect, this method based on artificial feature extraction has certain subjective and technical limitations, resulting in the recognition efficiency of the model cannot be improved.

In recent years, due to the continuous development of artificial intelligence, the types of data that need to be processed are more diversified, and image, as a kind of visual data, has a large number of processing needs, so deep learning with a more efficient feature extraction method has been proposed. Among them, convolutional neural network (CNN), as a direction of deep learning, is widely used in the field of agricultural engineering, such as food disease recognition, fruit quality detection and so on. CNN has been successfully applied to the detection and recognition of objects and regions in images. CNN automatically extracts features from images, video and audio to reduce the impact of human factors on the recognition effect. Compared with artificial feature extraction, this method not only saves time, but also increases the accuracy of model recognition. Zhang et al. (2020) extracted the texture, color and other characteristics of the fully convolutional network, and combined with hyperspectral technology to detect and segment the bruised tissue, unbruised tissue and calyx end of blueberries. The results showed that the method had excellent performance in the detection of early bruising of blueberries compared with the SVM. Parvathi and Selvi (2021) proposed an improved fast regional convolutional neural network model for mature coconut images in complex backgrounds, which together with residual networks (ResNet-50) improved detection values at two major maturity stages. The test results showed that the detection performance of the proposed method was higher and its classification loss rate was only 5.10%. Crr et al. (2020) proposed a small CNN model architecture to detect rice pests and diseases. The experiment results showed the average recognition rate can reach 93.30%, and its standard deviation was 0.96, which was smaller than other CNN models. Xie et al. (2021) proposed a lightweight model based on deep convolutional neural network (DCNN) for the recognition of external defect images of carrots. The recognition accuracy of the model in the test set was 97.04% and the detection speed of about 80 frames per second. Mkonyi L et al. and Qiu J et al. all used the method of transferring the pre-trained ImageNet model to manually fine-tune the number of fully connected layers neurons and learning rate of the classic VGG16 model, so as to modulate the identification accuracy of early tomato plants and rice leaf diseases. The recognition rate of the VGG16 proved that the model has the characteristics of high accuracy, strong generalization ability and small loss rate (Mkonyi et al., 2020; Qiu et al., 2021). A network was proposed by improving the basic learning rate of the GoogLeNet model, which recognized 8 kinds of corn disease images, and finally achieved the purpose of improving the recognition accuracy. It has been experimentally proved that, the average recognition accuracy reached 98.90% (Zhang et al., 2018b).

Although the image recognition and classification functions of deep neural networks are powerful, it is difficult to select suitable hyperparameters. CNN models rely on multiple hyperparameters (activation function, number of convolutional kernels, convolutional kernel size, learning rate, etc.). But its selection is often based on experience, and its selection rule is not clearly defined. If one of the selected hyperparameters are inappropriate, it will cause low model accuracy and large loss rate. Therefore, it is crucial to find a set of appropriate hyperparameters. For this reason, some researchers have used heuristic optimization algorithms to optimize the hyperparameters of CNN models, such as PSO, grey wolf optimization (GWO), and ant colony optimization (ACO) and so on. An adaptive cooperative PSO algorithm (ACPSO) was proposed, which took the output of the ACPSO algorithm as the weight of the multi-layer feed-forward network. In this way, the model training avoid falling into local optimal value, and effectively improved the recognition rate of CNN (Xiao et al., 2019). Tu et al. (2021) fused a Modified PSO algorithm (ModPSO), which made the structure of CNN not affected by the addition or elimination of the network layer. Compared to other algorithms, this algorithm can avoid falling into the situation of “precocious”. Kanwal et al. (2021) proposed a multi-objective PSO convolutional autoencoder by improving the speed and position update equations of particle individuals, making the method versatile and accurate. An enhanced GWO was proposed, which accelerated the convergence speed and improved the convergence rate compared with the classical GWO. Then the enhanced GWO was used to optimize the network topology and learning hyperparameters of CNN-LSTM. Experiments showed that this method can not only capture key features, but also encapsulate complex dependencies into time series tasks to perform time series tasks (Xie et al., 2020). Hyperspectral image analysis combined with a CNN model based on GWO optimization was often used in land cover classification, crop stage detection and other remote sensing aspects. GWO optimized six hyperparameters in CNN to make up for the shortcomings of traditional optimization methods, such as time-consuming and laborious. Experiments showed that the classification accuracy of the algorithm on the specified datasets was more than 99.00% (Ladi et al., 2022). The early diagnosis of Alzheimer’s disease based on CNN was proposed. ACO was used to optimize the hyperparameters in CNN, and the specific way was to back propagate the classification error in the iterative training of CNN model to the ACO, and finally obtained the CNN structure with the optimal combination of hyperparameters. This method was applied to Alzheimer’s disease neuroimaging initiative dataset by researchers, and the classification accuracy can reach 98.67% (Singh and Janghel, 2022). Compared with other swarm intelligence optimization algorithms, the whale optimization algorithm (WOA) used in this paper is easy to implement, has fast convergence speed, high convergence accuracy, and is not easy to fall into the local optimal solution, so it is widely used in various fields.

WOA used in this paper and its improved algorithms have been applied to industry, engineering and other fields (Mirjalili and Lewis, 2016). In order to solve the problem of minimizing the sum of the energy consumption cost and the completion time cost of the workshop, the discrete whale optimization algorithm (DWOA) was proposed to solve the mathematical model. The scale of the solution was determined by the parallel calculation of the two sub-problems of job arrangement and speed selection, and the population was initialized with DWOA to improve the quality of the initial solution. The variable field search strategy was integrated into the algorithm, which improved the search ability of the algorithm (Jiang et al., 2019). The WOA used to optimize the hyperparameters of the SVM for detecting and classifying the multi-power quality events, which made the SVM have higher classification accuracy (Dash and Subudhi, 2019). An improved WOA used to optimize fault detection and diagnostics for sensorless brushless DC motors. The simulation results showed the improved diagnostic strategy of WOA is the most effective (Vanchinathan et al., 2021).

Since the hyperparameters of CNN models are usually selected manually without explicit specification, this selection method may lead to lower final classification accuracy of the model. The research direction of this paper is to use the optimization algorithm instead of manual selection to avoid the problem of huge computing parameters and serious preemption of computing resources during model operation. The first contribution of this paper is to propose WOACW. First, the initialization population is improved using adversarial learning, which greatly improves the algorithm’s convergence rate and computational accuracy. Secondly, the number of iterations of global exploration is increased by modifying the convergence factor, that is, the global exploration ability of the algorithm is enhanced. Then, the author introduces weights in the optimization stage of the algorithm, and the local search ability is continuously enhanced with the increase of the number of iterations by adjusting the step size of the algorithm. The convergence factor and weight effectively balance the global exploration ability and local search ability of the algorithm. The chaotic map is introduced in it to enhance the robustness of the algorithm. Finally, the polynomial mutation operator is used after each iteration, and the mutation vector with a small probability further avoids the situation that the algorithm falls into the local optimal solution. Experiments show that WOACW has better optimization effect than WOA. The second contribution of this paper is to use WOACW’s strong optimization ability and fast convergence speed to optimize the hyperparameters of the lightweight CNN model, and identify them due to the identification of rice leaf disease images. The experiment verifies the feasibility of the method and provides an effective idea for the optimization of the CNN model.

The following parts of this paper are organized as follows. In the Section 2 of this paper, an improved whale optimization algorithm is proposed based on nonlinear convergence factor and weight cooperative self-mapping chaotic perturbation (WOACW). Simulation experiments show that WOACW has faster convergence speed and higher convergence accuracy than other WOAs. In the Section 3 of this paper, firstly, a simple CNN model is proposed, and then five common images of rice leaf diseases and healthy leaves were identified and classified using SimpleNet optimized by WOACW. The Section 4 of this paper is the conclusion and prospects, the research direction of subsequent experimental improvement and application of rice disease identification are indicated. These parts will be described in detail below.

## 2 Methods

At present, swarm intelligence optimization algorithms are widely used in many fields such as artificial intelligence and they are one of the key steps to solve complex problems. Inspired by the hunting behavior of whale populations, the whale optimization algorithm was proposed by Australian scholar Mirjalili in 2016, which had a simple structure, strong optimization ability, fast convergence speed and easy to implement. But at the same time there are problems such as low convergence accuracy and easy to fall into “precocious” (Kong et al., 2020). In order to solve such problems, many variants of the WOAs have been proposed. Guo et al. (2017) proposed the WOAWC, where the Cauchy inverse cumulative distribution function method and the adaptive weight method were used to improve the global and local search capabilities of the WOA, thus improving the convergence accuracy of the algorithm. Kong et al. (2020) proposed the AWOA, which used the adaptive adjustment weight method to improve the search ability of the algorithm in different iteration periods. The same time, they used the adaptive adjustment search strategy to increase the diversity of the population. Wang et al. (2019) proposed the CWOA, which used a chaotic reverse learning strategy to initialize the population, and adjusted the convergence accuracy and robustness of the population by cooperating with the weight with the nonlinear convergence factor function with chaos mapping. Huang et al. (2020) proposed the CPWOA, which optimized the algorithm by nonlinear convergence factor and weight. In order to explore the whole space more fully, the authors added a variation algorithm to the algorithm, which largely maintained the diversity of the population.

### 2.1 Whale optimization algorithm

Some areas of the whale’s brain have cells like humans, which can think, learn, and judge. The WOA simulates the group feeding activities of humpback whales. And the algorithm is divided into three stages: encircling prey, bubble-net attacking method and global search for prey. These three stages are described in detail below.

#### 2.1.1 Encircling prey

Due to the exact position of the whales for their prey during predation is unknown, the WOA assumes that the current optimal solution is the position closest to the target prey. After defining the optimal position, the other whales attempt to update their respective positions towards the optimal vector and gradually surround the best solution. The position update equation in the encircling prey phase is shown in Eq. 1:


 (1) $$X\left(t+1\right)=X^{*}\left(t\right)-A\cdot D$$

(2) $$D=\left|C\cdot X^{*}\left(t\right)-X\left(t\right)\right|$$

where *X*(*t*) represents the vector of the current whale’s location, X^*^(*t*) represents the current optimal position vector of whale position, *t* is the current number of iterations, *A* and *C* are learning factors. Note that *A* and *C* are derived from the following equations:


(3) $$A=2a\times r_{1}-a$$

(4) $$C=2\times r_{2}$$

(5) $$a=2-2\times t/T_{max}$$

where *a* is the convergence factor, which drops linearly from 2 to 0, *r*_1_ and *r*_2_ are random numbers between [0,1] , *t* is the current number of iterations, and *T*_*max*_ is the maximum number of iterations.

#### 2.1.2 Bubble-net attacking method

Spatially, whales follow a spiral. Therefore, the method first calculates the distance *D* between the whale’s position *X* and the position of the prey X^*^ (the current optimal solution). Then a spiral equation between the whale and its prey is established to simulate the whale’s spiral trajectory. The location update is shown in Eq. 6:


(6) $$X\left(t+1\right)=X^{*}\left(t\right)+D^{\prime \prime }\cdot e^{bl}\cdot \text{cos }\left(2\pi l\right)$$

(7) $$D^{\prime \prime }=|X^{*}(t)-X(t)|$$

where *D*″ represents the distance between the i^th^ whale and the current optimal solution, *b* is the constant which defines the shape of the logarithmic spiral, *l* is a random value within [−1,1].

To sum up, whales approach their prey in both encircling prey and bubble-net attacking method ways. To achieve synchronization of the model, the same probability *p* is chosen to select the hunting method which is shown in Eq. 8:


(8) $$X\left(t+1\right)=\{\begin{array}{cc} X^{*}\left(t\right)-A\cdot D & p<0.5\\ X^{*}\left(t\right)+D^{\prime \prime }\cdot e^{bl}\cdot \text{cos }\left(2\pi l\right) & p\geq 0.5 \end{array}$$

where *p* is a random number within [0,1].

#### 2.1.3 Global search for prey

The WOA randomly selects individual whales as the global optimal solution on a global scale, and other whale individuals are gathered, which enhances the global search capability of the algorithm. Its position update is shown in Eq. 9:


(9) $$X\left(t+1\right)=X_{rand}\left(t\right)-A\cdot D^{\prime }$$

(10) $$D\prime \;=\;|C\;\cdot \;X_{rand}(t)\;-X(t)|$$

where *X*_rand_(*t*)  represents the location of a randomly selected whale.

When *p*≥0.5 , the algorithm adopts the spiral surrounding method, as shown in Eq. 6. When *p*<0.5 , it includes two stages of global random exploration and local surrounding predation, and uses the |*A*| to take the random values. When |*A*|<1 , the algorithm adopts the local encircling prey phase, as shown in Eq. 1. When |*A*|≥1 , the algorithm employs the global search phase, as shown in Eq. 9.

### 2.2 WOACW

#### 2.2.1 Population initialization based on opposition-based learning

The recent studies have shown that the degree of population initialization is directly related to the convergence rate and computational accuracy of the algorithm (Bangyal et al., 2021), and good initialization of the population is helpful in improving the performance of the algorithm. However, the WOA often randomly selects values in the value range when initializing the population, which may cause the population to be unevenly distributed in space and affect the convergence efficiency of the entire algorithm. In recent years, the opposition-based learning strategies have been widely used to guide populations for approximate global optimal solutions (Ding et al., 2019). And it has been widely used in group intelligence algorithms such as PSO algorithm and butterfly optimization algorithm(BOA) (Agarwal and Srivastava, 2021; Guo et al., 2021) Therefore, this paper applies the opposition-based learning strategy to the WOA for population initialization, so that to improve the efficiency of the algorithm.

Assumed that the number of individual populations is *N* . The dimension of each individual is *D* . *x*_*id*_ exists in [*lb*_*d*_,*ub*_*d*_] , where *lb*_*d*_ and *ub*_*d*_ is the lower and upper bounds of the *d*^*th*^ dimensionvalue of the *i*^*th*^ individual vector, respectively. The initial population of the WOA is *X*={*x*_*i*_}(*i*=1,2,…,*N*) , where *x*_*i*_={*x*_*id*_}(*d*=1,2,…,*D*) . The value of the *d*^*th*^ dimension in the opposing population is shown in Eq. 11:


(11) $$x_{id}^{‘}=lb_{d}+ub_{d}-x_{id}$$

Use the above equation to generate the opposing populations *X*^′^ with the numbers of *n* . And $X^{\prime }=\left\{x_{i}^{\prime }\right\}\left(i=1,2,\ldots ,N\right)$ , where $x_{i}^{\prime }=\left\{x_{id}^{\prime }\right\}\left(d=1,2,\ldots ,D\right)$ . Subsequently, the random population *X* is merged with the opposing population *X*^′^ to get a new population {*X*∪^​^*X*^"^} . Finally, the fitness value of each individual in the new population is calculated and sorted. And the first *N* vectors with the best fitness are selected as the initial population *X*_*init*_ of the whole algorithm.

#### 2.2.2 Self-mapping chaotic nonlinear convergence factors and weights

Like other swarm intelligence optimization algorithms, the overall algorithm is consisting of the global exploration phase and the local exploitation phase. In the classical WOA, the convergence factor *a* decreases linearly from 2 to 0, so as to controlling the change of the parameter A (Eq. 3), thereby coordinating the global exploration phase and the local exploitation phase. For multi-objective problems, the solution vector should be selected more extensively in the value interval to avoid falling into the local optimal solution, in the period of global exploration. In the local exploitation phase, some better vectors obtained in the previous stage are quickly converged for saving calculation time. However, the linear convergence factor cannot balance the two phases well, so the linear convergence factor function needs to be changed to a nonlinear function, as shown in Eq. 12:


(12) $$a=2\cdot \text{cos }\left(\frac{\pi }{2}\cdot \frac{t}{T_{max}}\right)$$

After this, the number of iterations for global explorations is increased, making global exploration more sufficient.

This paper also introduces a chaotic sequences (Liu and Ye, 2011), which together with the nonlinear convergence factor function form a new convergence factor function, as shown in Eq. 13:


(13) $$a=a\cdot \left|y^{t}\right|$$

where *y*^0^ is a random number in (-1,1), and *y*^*t*^=1−2(*y*^*t*−1^)^2^ (*y*^*t*^∈(−1,1)) is a chaotic sequence generated from the self-logical mapping function.

The improved convergence factor function proposed above can well balance the global exploration ability and the local search ability. In the early stage of algorithm iteration, the global exploration ability is enhanced, but the convergence speed is slower. In the later stage of algorithm iteration, the algorithm convergence speed is too fast, leading to fall into local optimization. Therefore, the speed of the global exploration should be accelerated, and a subtle perturbation mechanism should be added to the local exploration period, thereby enhancing the robustness of the algorithm. In this paper, the weight *w*_1_ and *w*_2_ are added to the WOA, as shown in Eq. 14 and Eq. 15.


(14) $$w_{1}=\frac{\left(cos\;\left(\pi \cdot \frac{t}{T_{max}}\right)+1\right)}{2}$$

(15) $$w_{2}=w_{1}\cdot \left|y^{t}\right|$$

Through the improvement of the self-mapping chaotic nonlinear convergence factor and weight, the encircling prey, bubble-net attaching method and global search for prey are updated as Eq. 16, Eq. 17 and Eq. 18, respectively.


(16) $$X\left(t+1\right)=X^{*}\left(t\right)\cdot w_{2}-A\cdot D$$

(17) $$X\left(t+1\right)=X^{*}\left(t\right)\cdot w_{2}+D^{\prime \prime }\cdot e^{bl}\cdot \text{cos }\left(2\pi l\right)$$

(18) $$X(t+1)=X_{rand}(t)\cdot w_{1}-A\cdot D^{'}$$

#### 2.2.3 Polynomial mutation operator

Theoretically, all vectors during iteration gradually move closer to the optimal vector, and the algorithm can better determine which regions of the parameter space are worth exploring and calculating. However, due to the complexity of multi-objective functions, convergence accuracy cannot be guaranteed. Therefore, this paper uses the mutation operator (Alawad and Abed-alguni, 2022) to find the better solution may exist in the search space, ensuring the diversity of algorithms. The polynomial mutation referenced in this paper is shown in Eq. 19:


(19) $$X_{k}^{‘}=X_{k}+\delta \cdot \left(ub_{k}-lb_{k}\right)$$

(20) $$\delta =\{\begin{array}{cc} {\left[2u+\left(1-2u\right)1-\delta_{1}^{\mu_{m}+1}\right]}^{\frac{1}{\mu_{m}+1}}-1 & u\leq 0.5\\ 1-{\left[2\left(1-u\right)+2\left(u-0.5\right){\left(1-\delta_{2}\right)}^{\mu_{m}+1}\right]}^{\frac{1}{\mu_{m}+1}} & u>0.5 \end{array}$$

where $X_{k}^{\prime }$ is the optimal individual vector after mutation. *X*_*k*_ is the local optimal vector after each iteration. *D* is the maximum dimension of the vector, and *k*∈(1,2,…,*D*) .*ub*_*k*_ and *lb*_*k*_ are the upper and lower bounds of the *k*^*th*^ dimension, and *u* is a random number in [0,1] . *μ*_*m*_ represents the distribution index. *δ*_1_=(*X*_*k*_−*lb*_*k*_)/(*ub*_*k*_−*lb*_*k*_) , *δ*_2_=(*ub*_*k*_−*X*_*k*_)/(*ub*_*k*_−*lb*_*k*_).

Note that the greedy mechanism is used in each iteration. When the adaptability value of the mutated solution vector is better than the fitness value of the local optimal solution vector, the local optimal solution vector is replaced with the mutated solution. The output vector using polynomial variation is better than the current global optimal vector, which improves the convergence accuracy of the algorithm.

#### 2.2.4 Algorithm flow

To sum up, the WOACW is designed, and the flow is shown in **Table 1**.

**Table 1:** Pseudo code for the WOACW.

WOACW
begin
1: Initialization of population size *N*, number of iterations *T* and problem dimension *D*;
2: Generate an initial population with N individuals according to Section 2.2.1;
3: Record the current optimal vector and optimal fitness value;
4: Initialize *y*_0_, *μ*_*m*_;
5: while (*t*<*T*) do
6: Calculated the *y*_*t*_ sequence and a according to Eq. 13;
7: Calculated the *w*_1_ and *w*_2_ according to Eq. 14 and Eq. 15;
8: for *i*=1 to *N* do
9: Update parameters *A*, *D*, *p*, *l*;
10: for *j*=1 to *D* do
11: if (*p* < 0.5) do
12: if (|*A*| >= 1) do
13: Update the current individual position according to Eq. 18;
14: else if (|*A*| < 1) do
15: Update the current individual position according to Eq. 16;
16: end if
17: else if (*p* >= 0.5) do
18: Update the current individual position according to Eq. 17;
19: end if
20: end for
21: Update the optimal solution and the optimal vector;
22: Polynomial mutation according to Eq. 19 produces new variables;
23: Use the greed mechanism to preserve the optimal solution for this iteration;
24: end for
25: *t*=*t*+1;
26: end while
**end**

### 2.3 Algorithm performance analysis

The proponents of WOA algorithm compared WOA with other optimization algorithms (Mirjalili and Lewis, 2016). In the simulation test of this paper, we focus on the analysis of WOACW and other improved WOA. All simulation experiments were run on a computer with AMD R5-5600, 16G memory, 2.30GHz. And the program is programmed using MATLAB R2021b programming. In this paper, eight benchmark functions are selected to test the performance of the WOACW algorithm, which are shown in **Table 2**. The test functions are given by Eq. 21 ∼ Eq. 28. Among them, *f*_1_(*x*)∼*f*_5_(*x*) are unimodal benchmark functions, which mainly investigate the convergence rate and solution accuracy of the algorithm. *f*_6_(*x*)∼*f*_8_(*x*) are multimodal function, which mainly examines the comprehensive optimization ability of the algorithm.


(21) $$Sphere\;:\;f_{1}(x)=\overset{n}{\underset{i=1}{\sum }}x_{i}^{2}$$

(22) $$Schwefel\;1.2\;:\;f_{2}(x)=\overset{n}{\underset{i=1}{\sum }}{\left(\overset{i}{\underset{j-1}{\sum }}x_{j}\right)}^{2}$$

(23) $$Rosenbrock\;:\;f_{3}(x)=\overset{n-1}{\underset{i=1}{\sum }}\left[100{\left(x_{i+1}-x_{i}^{2}\right)}^{2}+{\left(x_{i}-1\right)}^{2}\right]$$

(24) $$Step\;:\;f_{4}(x)=\overset{n}{\underset{i=1}{\sum }}{\left(\left[x_{i}+0.5\right]\right)}^{2}$$

(25) $$Quartic\;:\;f_{5}(x)=\overset{n}{\underset{i=1}{\sum }}ix_{i}^{4}+random\left[0,1\right)$$

(26) $$\begin{array}{c} Penalized\;1:\;f_{6}(x)=\frac{\pi }{n}\{10\text{sin}(\pi y_{1})+\sum_{i=1}^{n-1}{\left(y_{i}-1\right)}^{2}\left[1+10\overset{2}{\sin}(\pi y_{i+1})\right]\}+\sum_{i=1}^{n}u(x_{i},10,100,4),\\ y_{i}=1+\frac{x_{i}+1}{4},u(x_{i},a,k,m)=\{\begin{array}{ll} k{\left(x_{i}-a\right)}^{m} & x_{i}>a\\ 0 & -a<x_{i}<a\\ k{\left(-x_{i}-a\right)}^{m} & x_{i}<-a \end{array} \end{array}$$

(27) $$Penalized\;2\;:f_{7}(x)=0.1\{\overset{2}{\sin}(3\pi x_{1})+\sum_{i=1}^{n}{\left(x_{i}-1\right)}^{2}\left[1+\overset{2}{\sin}(3\pi x_{1}+1)\right]+{\left(x_{n}-1\right)}^{2}\left[1+\overset{2}{\sin}(2\pi x_{n})\right]+\sum_{i=1}^{n}u(x_{i},5,100,4)\}$$

(28) $$Kowalik\;:\;f_{8}(x)=\overset{11}{\underset{i=1}{\sum }}{\left[a_{i}-\frac{x_{1}(b_{i}^{2}+b_{i}x_{2})}{b_{i}^{2}+b_{i}x_{3}+x_{4}}\right]}^{2}\;$$

**Table 2:** Eight benchmark functions.

Function	Name	Dimension	Search scope	Optimal value
*f*_1_(*x*)	Sphere	30	[−100,100]	0
*f*_2_(*x*)	Schwefel 1.2	30	[−100,100]	0
*f*_3_(*x*)	Rosenbrock	30	[−30,30]	0
*f*_4_(*x*)	Step	30	[−100,100]	0
*f*_5_(*x*)	Quartic	30	[−1.28,1.28]	0
*f*_6_(*x*)	Penalized 1	30	[−50,50]	0
*f*_7_(*x*)	Penalized 2	30	[−50,50]	0
*f*_8_(*x*)	Kowalik	4	[−5,5]	3.00e-04

In order to test the optimization ability of the WOACW, the WOA and four improved WOAs are used for comparison, including the CWO, the CPWOA, the WOAWC and the AWOA. The eight benchmark functions in **Table 2** are optimized and solved, and the experimental parameters of the six WOAs are set as shown in **Table 3**.

**Table 3:** Experimental parameters of the six WOAs.

Objects	Values of parameters
Common part	*N* = 30, *T* = 500, *Dim* = 30, *b* = 1
WOA	–
CWOA	*a*_*initial*_ = 2, *a*_*final*_ = 0, *w*_*initial*_ = 0.9, *w*_*final*_ = 0.2
CPWOA	*μ*_*m*_ = 2
WOAWC	–
AWOA	*d*_1_ = 1.0e-4, *d*_2_ = 1.0e-4
WOACW	*μ*_*m*_ = 2

Note that if a is no redefinition in the above algorithm, the corresponding algorithm is linearly reduced from 2 to 0.

In this paper, two evaluation metrics are used: the optimal accuracy average (Ave) and the optimal accuracy standard deviation (Std), where the average reflects the accuracy of the algorithm and the standard deviation reflects the stability of the algorithm solution. In order to eliminate the randomness of the algorithm, 30 independent experiments are carried out on 6 WOAs. The experimental results are shown in **Table 4**.

**Table 4:** Performance comparison of eight WOAs.

Function	Index	WOA	CWOA	CPWOA	WOAWC	AWOA	WOACW
*f*_1_(*x*)	Ave	9.28e-34	**0**	1.66e-31	**0**	**0**	**0**
	Std	5.08e-33	**0**	6.49e-31	**0**	**0**	**0**
*f*_2_(*x*)	Ave	4.65e+04	**0**	6.60e+04	**0**	**0**	**0**
	Std	1.44e+04	**0**	1.39e+04	**0**	**0**	**0**
*f*_3_(*x*)	Ave	2.81e+01	2.80e+01	5.87e+00	2.86e+01	2.79e+01	**1.25e+00**
	Std	4.18e-01	3.00e-01	7.41e+00	7.29e-02	3.30e+00	**2.28e-02**
*f*_4_(*x*)	Ave	4.34e-01	7.85e-01	**1.22e-01**	3.05e-01	4.95e-01	2.31e-01
	Std	2.30e-01	3.34e-01	4.85e-02	6.88e-02	1.98e-01	**1.16e-02**
*f*_5_(*x*)	Ave	2.57e-03	1.18e-04	4.26e-03	1.35e-04	1.20e-04	**4.62e-05**
	Std	2.83e-03	8.97e-05	5.04e-03	9.68e-05	6.94e-05	**3.65e-05**
*f*_6_(*x*)	Ave	2.38e-02	3.99e-02	1.13e-02	1.84e-02	2.29e-02	**1.09e-02**
	Std	1.07e-02	1.81e-02	9.97e-03	1.87e-02	1.03e-02	**5.69e-03**
*f*_7_(*x*)	Ave	5.75e-01	5.58e-01	**1.67e-01**	2.26e-01	2.25e-01	1.88e-01
	Std	2.75e-01	1.84e-01	8.78e-02	4.59e-02	1.28e-01	**1.08e-02**
*f*_8_(*x*)	Ave	1.04e-03	6.14e-04	1.06e-03	4.25e-04	3.94e-04	**3.78e-04**
	Std	8.25e-04	1.91e-04	7.85e-04	1.05e-04	**5.34e-05**	6.99e-05

Note that the bold numbers indicate the better performance.

**Figure 1:**
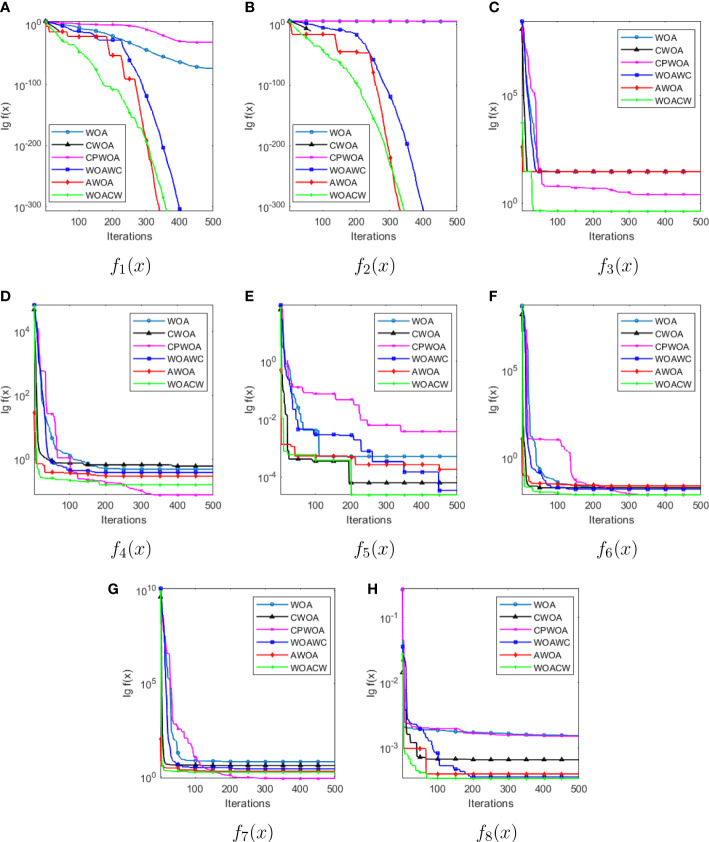
The convergence curves of WOA, CWOA, CPWOA, WOACW, AWOA and WOACW to f1(x)~f8(x) benchmark functions (as shown in sub-figure **A–H** respectively).

For solving unimodal benchmark functions, we can see that the WOAWC, the AWOA and the WOACW all converge to 0 in *f*_1_(*x*) and *f*_2_(*x*) functions, which is the theoretical minimum. After *f*_3_(*x*) and *f*_5_(*x*) function testing, the WOACW convergence accuracy is optimal. After the 6 algorithms have been tested as functions *f*_4_(*x*) , although the convergence accuracy of the CPWOA is better than that of the WOACW, the standard deviation of the WOACW is smaller and more robust. For solving multimodal test functions, the algorithms optimize the *f*_6_(*x*) and *f*_8_(*x*) functions, and the average convergence accuracy of the WOACW is smaller than that of the other five algorithms. However, after testing the *f*_7_(*x*) , it is found that the convergence accuracy of the WOACW is not optimal, second only to the CPWOA. The reason is that the function has many local minimums, it is difficult to detect the global optimal solution, resulting in low convergence accuracy. In summary, the optimization capability of the WOACW is superior to several other WOAs. Although the convergence performance of the WOACW is not as good as other algorithms in individual test functions, it is still at the forefront of performance ranking and shows sufficient competitiveness. In order to clearly observe the curve change of the convergence functions, this paper takes the constant logarithmic function when plotting, and the convergence curves of eight benchmark functions are shown in **Figure 1**.

## 3 Results and discussion

### 3.1 CNN and WOACW_SimpleNet algorithm thought

**Figure 2:**
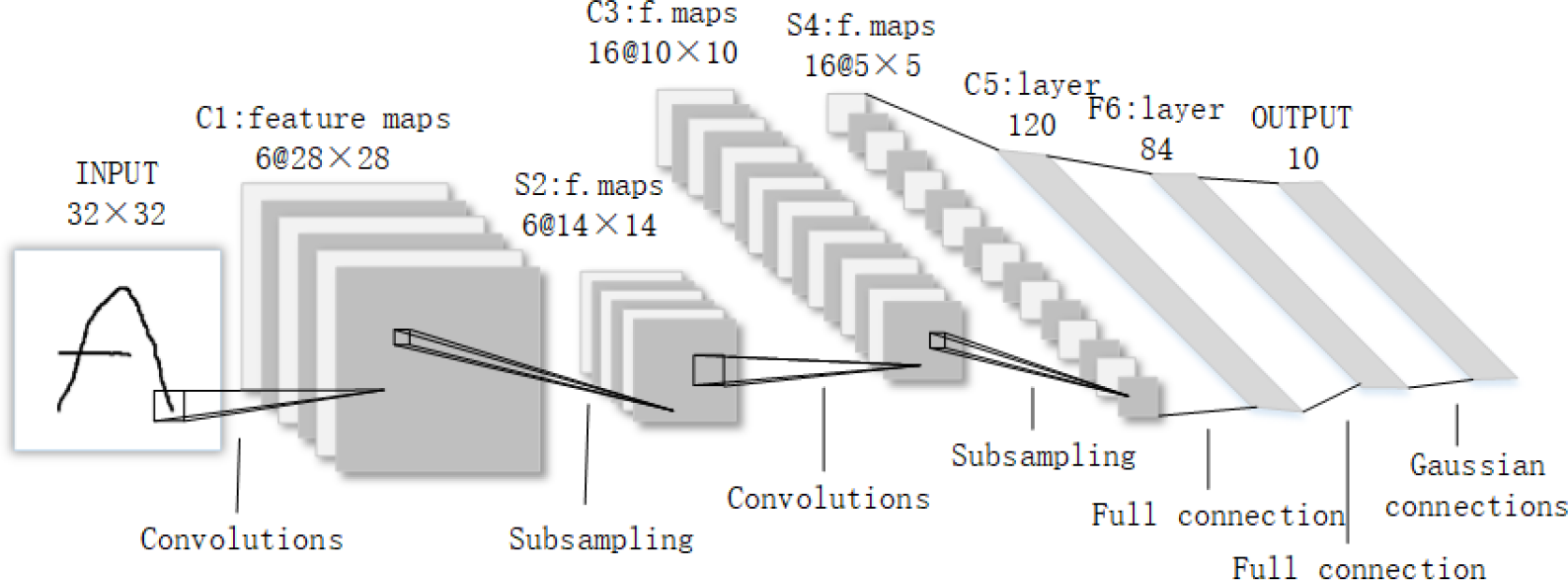
Schematic diagram of LeNet-5 structure.

Compared with the manual feature extraction method, CNN uses automatic feature extraction, and its error feedforward function enables it to identify and classify targets with higher accuracy. At present, it has achieved great success in the fields of image processing (Öztürk et al., 2018), object detection (Kumar and Srivastava, 2020) and face recognition (Khan et al., 2019). The LeNet-5 model was first proposed as a convolutional neural network. Its structure is not complex, the number of layers is small, mainly including convolutional layers, pooled layers and fully connected layers. The alternating settings of convolutional and pooling layers in the model can abstract the input images into a set of feature maps through multiple nonlinear transformations, then the neurons of the fully connected layers are used to classify these features. The network structure of the LeNet-5 model is shown in **Figure 2**.

The first core part of the CNN is the convolutional layer, which performs convolutional calculations on the input data so that the complex features of the image can be fully described. The correspondence between the input and output of the convolutional layer is shown in Eq. 29.


(29) $$x_{i}^{out}=f_{cov}\left(\overset{n}{\underset{i=1}{\sum }}x_{i}^{in}\times w_{ij}+b_{j}\right)$$

where $x_{i}^{out}$ represents the output of the neuron, *f*_*cov*_(·) is the activation function of current convolutional layer, of which the ReLU activation function is used in LeNet-5, *n* input signals can input neurons *j*_*th*_ at the same time, $x_{i}^{in}$ represents the input signal, *w*_*ij*_ represents weights to connect the $x_{i}^{in}$ and neuron *j* , *b*_*j*_ is the bias value of the network.

The second core part of the CNN is the pooling layer, which usually lies after the convolutional layer. It is used to reduce the size of the feature map, thereby reducing the number of parameters and retaining the data information as much as possible. The correspondence of the pooling layer is shown in Eq. 30:


(30) $$t_{i}^{out}=f_{sub}\left(t_{j}^{in},t_{j+1}^{in}\right)$$

where $t_{i}^{out}$ represents the output value of the pooling layer, and *f*_*sub*_(·) is a pooling type function, which can be maximize pooling or average pooling. $t_{j}^{in}$ represents the output value of the *j*^*th*^ neuron of the pool layer corresponding to the input characteristic plane.

The CNN reduces the number of parameters in the model computation by combining local awareness, weight sharing and pooling techniques. However, the hyperparameters such as the number of convolutional kernels, the size of convolutional kernels and the learning rate are obtained by researchers through many experiments, which increase the time cost, but also usually cannot obtain the optimal parameter combination, resulting in the low model training accuracy. Therefore, this paper uses the WOACW to optimize the hyperparameter of the CNN. In order to verify the feasibility of the WOACW_SimpleNet, the optimized hyperparameter is used to improve the accuracy of the simple CNN model for image recognition of rice leaf diseases.

**Figure 3:**
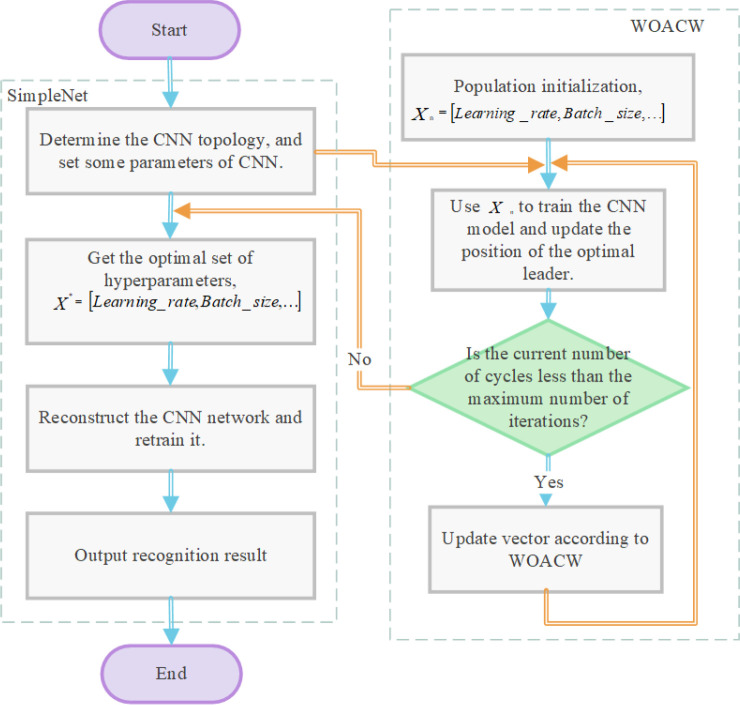
WOACW_SimpleNet flowchart.

The WOACW has few tunable parameters and has fast convergence speed. It takes the cross-entropy loss function as the objective function of WOACW and the cross-entropy loss value of each iteration test as the fitness function value. The cross-entropy loss function equation is shown in Eq. 31.


(31) $$C=-\frac{1}{n}\sum_{x}\left[yln\left(\hat{y}\right)+\left(1-y\right)ln\left(1-\hat{y}\right)\right]$$

where *n* is the total number of training data, *x* is the training input, $\hat{y}$ is the actual output, and *y* is the corresponding target output. The optimal solution vector obtained by the algorithm is used as a set of hyperparameters of the optimal CNN, and then reconstructs the CNN structure. The WOACW_SimpleNet flowchart is shown in **Figure 3**. For ease of programming, the concept of “vector” is transformed into “list” in Python, so that the index value of the elements in the vector to the list can be input into CNN through the index.

### 3.2 Image acquisition and preprocessing

In this experiment, images of healthy leaves and five common leaf diseases of rice are collected. The images are derived from the public dataset of Kaggle website and the experimental field of Bayi Agricultural University in Heilongjiang. The shooting time was June and July 2021. In the manual acquisition process, considering the impact of light on the image acquisition, the sampling time of the image was set to 7:00-9:00 and 16:00-18:00. The equipment used for image acquisition is Huawei nova7 smart phone, and the pixels of its rear camera are 64.0 million. In order to obtain a relatively large and clear picture of the disease spots in the picture, the distance between the camera and the rice leaf spots was about 0.25 m during the shooting process. In the end, 634 leaf pictures of three-channel rice were collected, where 630 valid samples were collected, including rice blast, bacterial leaf blight, brown spot disease, sheath blight, tungro disease and healthy leaf images, each in the image format JPG.

**Figure 4:**
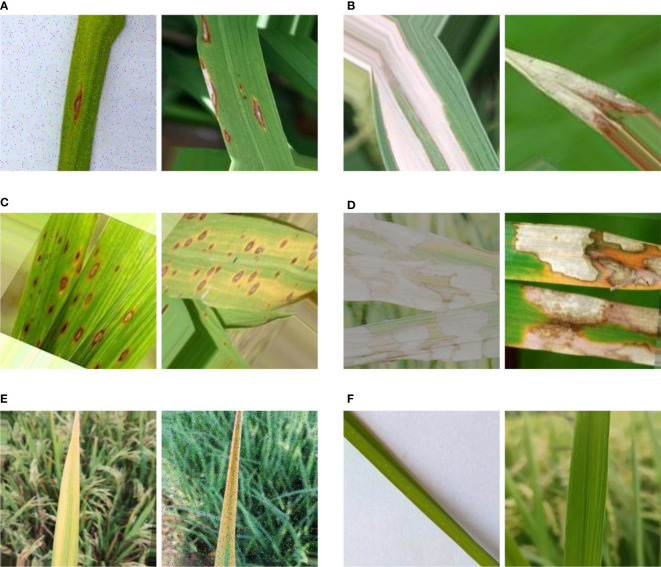
Rice leaf disease images. Note that **(A)** stands for rice blast, **(B)** stands for bacterial leaf blight, **(C)** stands for brown spot disease, **(D)** stands for sheath blight, **(E)** stands for tungro disease and **(F)** stands for health.

The experimental environment of this paper is Windows 10, the processor is AMD R5-5600, and the memory is 16G. In order to unify the input dimensions of the deep learning model, all images in the dataset are scaled to 224 × 224 pixels. Because training CNN requires a lot of data, the images need to be expanded before the experiment. The specific data augmentation methods include geometric transformation, nonlinear transformation, Gaussian blur, salt and pepper noise, etc. The expanded rice leaf disease dataset has a total of 3060 images, which helps to reduce the overfitting in the training stage. Some rice leaf disease images after data augmentation are shown in **Figure 4**.

Rice blast is distributed in various rice regions throughout the country, mainly damaging leaves, stems and ears. Among them, leaf blast can occur in the whole growth period. The center of the disease spot is gray white, the edge is brown, there is a pale-yellow halo outside, and there is a gray mold layer on the back of the leaf. When the disease spots are more, they form irregular large spots and produce fewer spores. Bacterial leaf blight can occur in all organs during the whole growth period, and the leaves are the most susceptible to the disease. The disease starts from the leaf tip or the edge of the leaf. Dark green water soaked and linear spots appear at first, and soon yellow white disease spots are formed along the linear spots. Then, the disease spots expand along both sides of the leaf edge or the middle rib, and become yellowish brown, and finally become dry white. The boundary of the disease spots is obvious, and the disease spots are not convex. In the initial stage of rice infected with brown spot disease, it is a small brown water-soaked spot, and then it expands into a spindle shaped or irregular reddish-brown stripe, with yellow halo at the edge, grayish brown at the center of the disease spot, and the disease spot often melts into a large stripe, making the leaves locally gray and sterile (Azim et al., 2021). Rice sheath blight can occur from seedling stage to Panicle stage. When the leaves are infected, the disease spots are in the shape of clouds, and the edges fade to yellow. When the humidity of rice growing environment is high, white reticular hyphae grow at the disease site, and then converge into white hyphae, forming dark brown sclerotia, which is easy to fall off. Tungro disease of rice causes the affected plants to shrink and the leaves to change color, and the growth declines. The leaves are orange to yellow, mottled on the young leaves, and rusty spots on the old leaves.

### 3.3 CNN model structure for rice leaf disease identification

In the experiment, the Keras 2.6.0 deep learning framework is used. This paper constructs an 11-layer convolutional neural network model, consisting of four convolutional layers, four pooling layers and three fully connected layers. Compared with the VGG16 model and the InceptionV3 model, the structure of this 11-layer CNN model is relatively simple. The pooling type is the Max-pooling with step size of 2, which can condense the data features for reduce the number of parameters required for the subsequent layers. The nonlinear activation function used in convolutional layers and fully connected layers is ReLU, and every padding takes valid. Each layer uses the dropout, and 20% of neurons are randomly discarded to alleviate the degree of overfitting and underfitting, saving the model’s better prediction efficiency. The activation function of the output layer is Softmax.

Assuming that the population number is *M* , the number of iterations of the WOACW optimization is *N* , and the number of CNN model training times is epochs. That is, the time cost of parameter optimization is *M*×*N*×*epochs* . Considering the training cost, this paper set *M*=15 and *N*=30 . Initialize the position of the whale individual by using the hyperparameters in **Table 5**.

**Table 5:** Hyperparameters in the WOACW_SimpleNet.

Vector components	Hyperparameters	Range of initial values
*x*_1_	Number of convolutional kernels in *C*1	10−100(*Z*)
*x*_2_	Number of convolutional kernels in *C*2	10−100(*Z*)
*x*_3_	Number of convolutional kernels in *C*3	10−100(*Z*)
*x*_4_	Number of convolutional kernels in *C*4	10−100(*Z*)
*x*_5_	Size of convolutional kernels in *C*1	3×3, 5×5, 7×7, 9×9
*x*_6_	Size of convolutional kernels in *C*2	3×3, 5×5, 7×7, 9×9
*x*_7_	Size of convolutional kernels in *C*3	3×3, 5×5, 7×7, 9×9
*x*_8_	Size of convolutional kernels in *C*4	3×3, 5×5, 7×7, 9×9
*x*_9_	Learning rate	0.001−0.1(*Z*)
*x*_10_	Batch size	40−100(*Z*)

According to **Table 5**, the WOACW uses some of the hyperparameters as the population solution vectors. Activation functions and pooling types are not chosen for optimization because they are less selective and less effective. If the step size is optimized, the processed image size will be small. In this case, the CNN is not very effective at extracting local features, and there is no guarantee that there is a large search space for other parameters.

**Figure 5:**
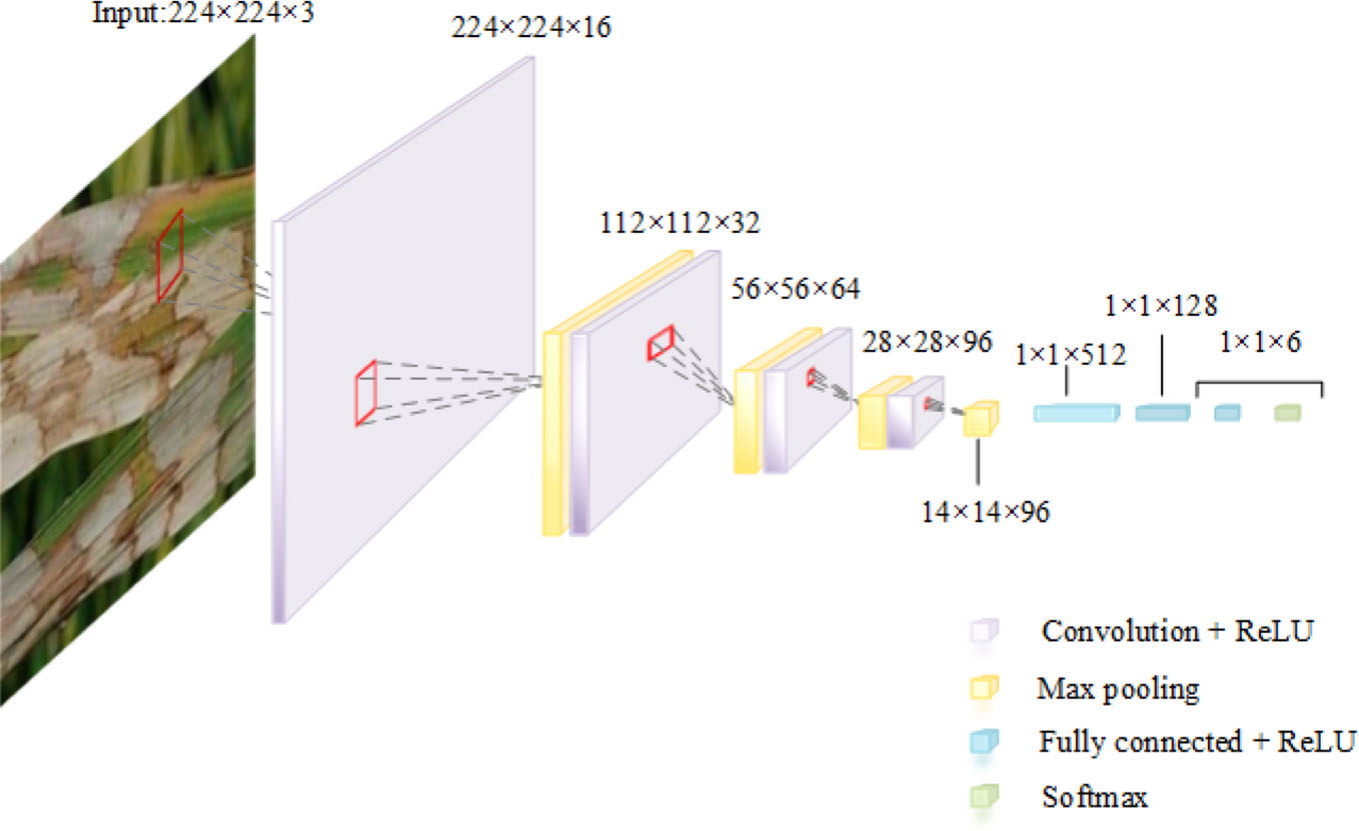
Schematic diagram of SimpleNet structure.

After 450 times of model training, the optimal combination of CNN parameters selected by the WOACW_SimpleNet is shown in **Table 6**. Additionally, the model structure diagram is shown in **Figure 5**.

**Table 6:** WOACW_SimpleNet optimal hyperparameters.

Vector components	Hyperparameters	Optimal value
*x*_1_	Number of convolutional kernels in *C*1	16
*x*_2_	Number of convolutional kernels in *C*2	32
*x*_3_	Number of convolutional kernels in *C*3	64
*x*_4_	Number of convolutional kernels in *C*4	96
*x*_5_	Size of convolutional kernels in *C*1	3×3
*x*_6_	Size of convolutional kernels in *C*2	3×3
*x*_7_	Size of convolutional kernels in *C*3	3×3
*x*_8_	Size of convolutional kernels in *C*4	3×3
*x*_9_	Learning rate	0.0052
*x*_10_	Batch size	58

**Figure 6:**
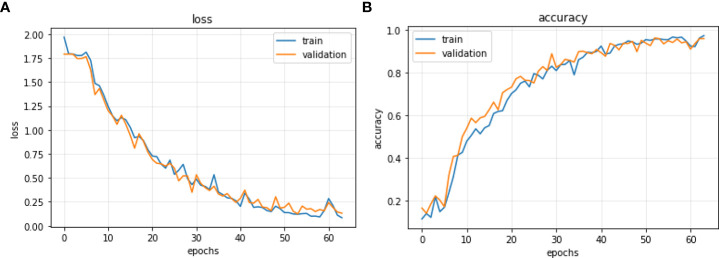
SimpleNet training curves. **(A)** The loss curves of SimpleNet. **(B)** The accuracy curves of SimpleNet.

The CNN model is reconstructed *via* the above optimal hyperparameters. And the ratio of images of training set, validation set and test set from the rice disease image dataset is 6:2:2. To solve the problem that the number of epochs needs to be set manually, the “early stopping” method is used to determine the number of trainings. When the validation loss is not reduced for 10 consecutive epochs, it is considered that the loss is no longer decreasing and the model stops training. Compared the loss rate and accuracy of the model on the training set and the validation set, as shown in **Figure 6**.

It is clear that the model with the above hyperparameters has good learning performance. In the first 50 iterations, the accuracy rate of the training set and the verification set rise rapidly, and the convergence curve fluctuates greatly. After 50 iterations, the convergence curve remains relatively stable. The accuracy rate keeps fluctuating and rising, at the same time the loss rate keeps fluctuating and falling. It shows that the model does not falling into underfitting and overfitting, and could continuously learn.

To illustrate the effectiveness of the SimpleNet, several classical convolutional models including the VGG16, the InceptionV3, and the MobileNetV2 are compared with the SimpleNet. Due to the smaller dataset used in this experiment, the transfer model training is performed by modifying the fully connected layer of these three models.

Evaluation metrics are accuracy, precision, recall, F1-score and training time for a single image, where the first four metrics are shown as Eq. 32 ∼ Eq. 35:


(32) $$Accuracy=\frac{TP+TN}{TP+FP+TN+FN}$$

(33) $$Precision=\frac{TP}{TP+FP}$$

(34) $$Recall=\frac{TP}{TP+FN}$$

(35) $$F1-score=\frac{2\times Precision\times Recall}{Precision+Recall}$$

where, *TP* is a class of positive samples predicted to be positive, *TN* is class of negative samples predicted to be negative, *FP* is a class of negative samples predicted to be positive, *FN* is a class of positive samples predicted to be negative.

The leaf features of the six kinds of rice leaf disease images used in this paper are identified by the VGG16, the MobileNetV2, the InceptionV3 and the SimpleNet, and the overall evaluation metrics are shown in **Table 7**. It can be seen from the table that although the training time of VGG16 and SimpleNet in a single picture is longer than that of the other two models, the average recognition accuracy of SimpleNet for rice leaf diseases is the highest, up to 99.35%, which is about 4-8 percentage points higher than that of other models. The SimpleNet outperformed the training results of the other three CNN models in terms of accuracy, recall, and F1-score, the reason is that difference between rice spots is small, and few information about the image characteristics of related spots. Since this paper studies specific rice leaf diseases, only the identification performance indicators of rice leaf disease images in the model are considered. Calculating a large number of features will cause the training effect of the model to be unsatisfactory. **Table 8** shows the evaluation metrics of the four CNN models for each rice leaf disease. Specify the labels ∈ {0, 1, 2, 3, 4, 5} correspond to the features ∈ {rice blast, bacterial leaf blight, brown spot disease, sheath blight, tungro disease, health}.

**Table 7:** The overall evaluation metrics of four CNN models for rice leaf diseases.

Model	Accuracy(%)	Precision(%)	Recall(%)	F1-score(%)	Training time(s)
VGG16	91.18	92.47	91.18	91.82	8.585
MobileNetV2	92.15	94.35	92.16	93.24	1.337
InceptionV3	95.26	96.08	95.26	95.67	1.974
SimpleNet	99.35	99.38	99.35	99.36	8.654

Note that the column name “Training time” refers to the training time for a single image.

**Table 8:** Evaluation metrics of four CNN models for six kinds of rice leaf diseases.

Model	Metrics	0	1	2	3	4	5
VGG16	Accuracy	0.928	0.833	0.931	0.947	0.928	0.904
	Precision	0.851	1.000	0.817	0.990	0.933	0.958
	Recall	0.951	0.716	0.961	1.000	0.951	0.892
	F1-score	0.898	0.834	0.883	0.995	0.942	0.924
MobileNetV3	Accuracy	0.855	0.953	0.863	0.953	0.953	0.953
	Precision	1.000	0.699	1.000	1.000	1.000	0.962
	Recall	0.755	1.000	0.775	1.000	1.000	1.000
	F1-score	0.860	0.823	0.873	1.000	1.000	0.981
InceptionV3	Accuracy	0.968	0.972	0.900	0.964	0.972	0.972
	Precision	0.990	0.803	1.000	1.000	1.000	0.971
	Recall	0.990	1.000	0.745	0.980	1.000	1.000
	F1-score	0.990	0.891	0.854	0.990	1.000	0.985
SimpleNet	Accuracy	0.988	0.996	0.992	0.992	0.996	0.997
	Precision	1.000	0.971	0.991	1.000	1.000	1.000
	Recall	0.980	1.000	0.990	0.990	1.000	1.000
	F1-score	0.990	0.985	0.990	0.995	1.000	1.000

**Figure 7:**
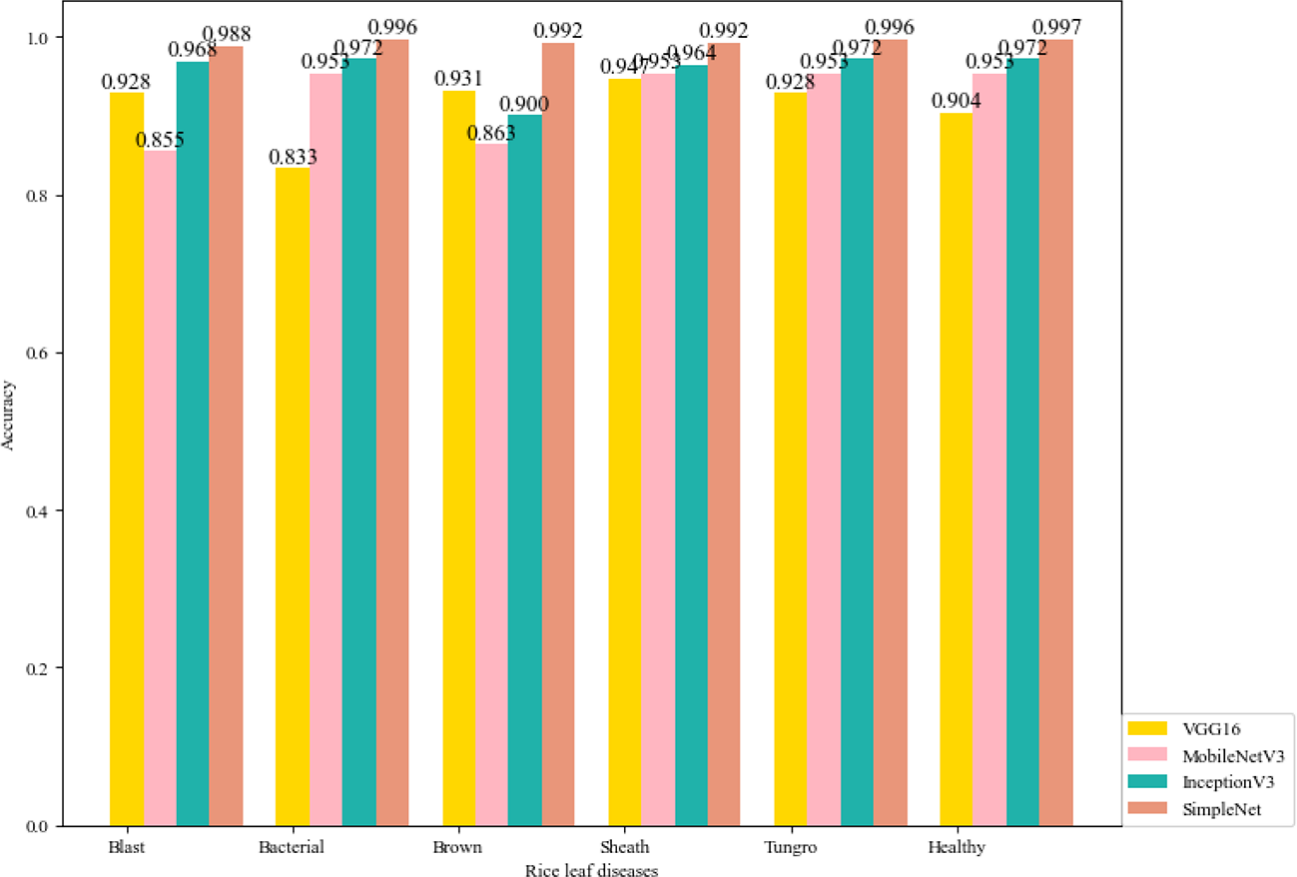
Recognition accuracy of four CNNs for the six kinds of rice leaf disease images.

In order to observe the recognition effect of each disease more intuitively in different models, the output visualization is shown in **Figure 7**. For each rice disease, the SimpleNet has the highest identification accuracy, reaching more than 99.00%. For the InceptionV3 model, the accuracy of the model in identifying brown spot disease is about 90.00%, but the accuracy of recognizing other types of diseases is more than 96.00%. The accuracy of the VGG16 model and the MobileNetV2 model are not stable, which indicates the generalization ability is poor. To sum up, the experiment results show that the SimpleNet model has good identification accuracy and better robustness for the six kinds of rice leaf diseases.

**Figure 8:**
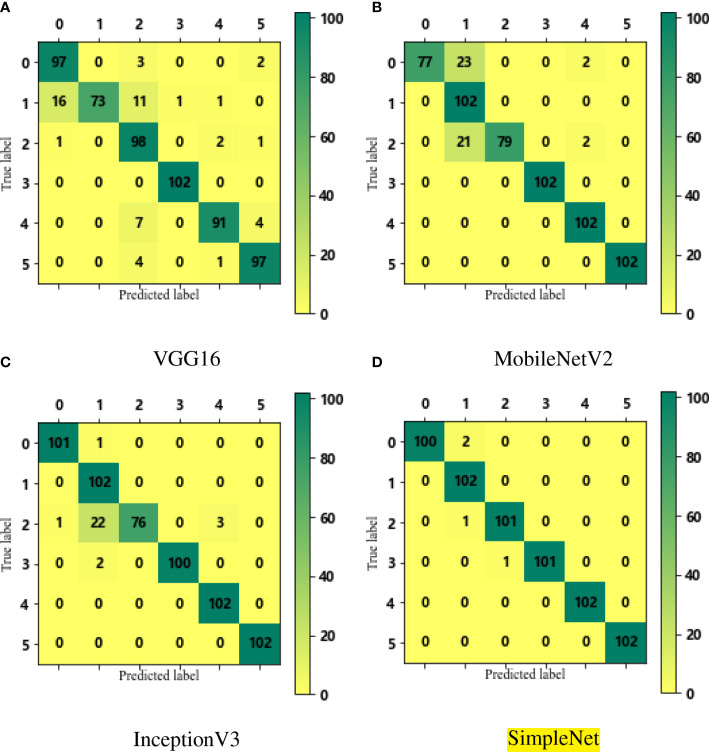
The confusion matrices for VGG16, MobileNetV2, Inception V3 and SimpleNet to identify six rice leaf diseases (as shown in sub-figure **A–D** respectively).

The confusion matrices of the four CNNs on the test set are shown in the **Figure 8**. The number of pictures of each disease on the test set is 102, and the prediction results of the diseases can be intuitively observed through color depth and numerical size in the confusion matrix. From the confusion matrix of the three transfer CNN models, some diseases such as rice blast, bacterial leaf blight and brown spot disease have a prediction bias of about 25.00%. In **Figure 8**(d), the SimpleNet misidentifies only 2.00% of rice blast leaves as bacterial leaf blight leaves, and the prediction of other leaf diseases is accurate. It can also be seen that the model is better at recognition and classification than the other three CNN models.

## 4 Conclusion

In this paper, the WOACW algorithm is proposed to optimize hyperparameters of CNNs to avoid the influence of human factors for detecting the rice disease. First, by simulating the values of the eight benchmark functions, the results show that WOACW is generally better than the WOA, the CWOA, the CPWOA, the WOAWC and the AWOA in terms of convergence precision and convergence rate. Secondly, a simple CNN model is built by stacking the convolutional layers and the pooling layers. Finally, the hyperparameters of the SimpleNet are optimized by WOACW using the rice leaf disease image in the experiment. It can be seen that SimpleNet has higher recognition accuracy than some classic CNNs, which can reach 99.35%.

Future work mainly includes the following three aspects. First, we will optimize the classical CNN models in the future work. The optimization direction includes deep structural parameters such as network structure and network weights, etc. Since the CNN models with complex structure generates a large number of computational parameters in the training process, we will use cloud servers and other machines with large computing capacity to train the model. The final goal of optimization is to optimize the model with better universality and model performance indicators, and increase the applicability of the model to the datasets with a large number of data characteristics. Second, the samples will be enriched in the future to further improve the identification accuracy and practical application value of the model. Third, the optimized convolutional model will be regarded as the core module for developing mobile applications, so that agricultural workers of different professional levels can directly identify and classify the rice leaf diseases.

## Data availability statement

The raw data supporting the conclusions of this article will be made available by the authors, without undue reservation.

## Author contributions

YL: Writing - Original Draft, Investigation, Software. XZ: Conceptualization, Supervision, Funding acquisition. NZ: Methodology, Writing - Review and Editing, Project administration. WL: Formal analysis, Visualization. RS: Validation. All authors contributed to the article and approved the submitted version.

## Funding

This work was supported in part by the National Natural Science Foundation of China under Grants U21A2019, 61873058, 61933007 and 62373271, the Hainan Province Science and Technology Special Fund under Grant ZDYF2022-SHFZ105, Heilongjiang Natural Science Foundation of China under Grant LH2020F042, the Scientific Research Starting Foundation for Post Doctor from Heilongjiang under Grant LBH-Q17134 and the Open Fund of the Key Laboratory for Metallurgical Equipment and Control of Ministry of Education in Wuhan University of Science and Technology under Grant 2018A02 and MECOF2019B02.

## Conflict of interest

The authors declare that the research was conducted in the absence of any commercial or financial relationships that could be construed as a potential conflict of interest.

## Publisher’s note

All claims expressed in this article are solely those of the authors and do not necessarily represent those of their affiliated organizations, or those of the publisher, the editors and the reviewers. Any product that may be evaluated in this article, or claim that may be made by its manufacturer, is not guaranteed or endorsed by the publisher.
